# AtPID: a genome-scale resource for genotype–phenotype associations in Arabidopsis

**DOI:** 10.1093/nar/gkw1029

**Published:** 2016-11-29

**Authors:** Qi Lv, Yiheng Lan, Yan Shi, Huan Wang, Xia Pan, Peng Li, Tieliu Shi

**Affiliations:** 1 Center for Bioinformatics and Computational Biology, and the Institute of Biomedical Sciences, School of Life Sciences, East China Normal University, Shanghai 200241, China; 2 School of Finance and Statistics, East China Normal University, Shanghai 200241, China

## Abstract

AtPID (*Arabidopsis thaliana* Protein Interactome Database, available at http://www.megabionet.org/atpid) is an integrated database resource for protein interaction network and functional annotation. In the past few years, we collected 5564 mutants with significant morphological alterations and manually curated them to 167 plant ontology (PO) morphology categories. These single/multiple-gene mutants were indexed and linked to 3919 genes. After integrated these genotype–phenotype associations with the comprehensive protein interaction network in AtPID, we developed a Naïve Bayes method and predicted 4457 novel high confidence gene-PO pairs with 1369 genes as the complement. Along with the accumulated novel data for protein interaction and functional annotation, and the updated visualization toolkits, we present a genome-scale resource for genotype–phenotype associations for Arabidopsis in AtPID 5.0. In our updated website, all the new genotype–phenotype associations from mutants, protein network, and the protein annotation information can be vividly displayed in a comprehensive network view, which will greatly enhance plant protein function and genotype–phenotype association studies in a systematical way.

## INTRODUCTION

Protein functional annotation and the protein networks including protein–protein interactions and regulatory relations are essential for understanding the underlying mechanism of the biological system. AtPID is a comprehensive data resource developed using *Arabidopsis thaliana* as the model system for protein interactions and functional annotation. From the year 2005, we started to collect the protein–protein interactions (PPIs) from literature and released AtPID 1.0, which only included limited curated PPIs and protein functional annotations from TAIR (The Arabidopsis Information Resource) (1). Due to the increasing demand of the comprehensive PPI from related research communities, we extended the PPI network by different computational methods and released AtPID 2.0 in 2006. In order to further increase the coverage and overcome the false positive issue within the predicted dataset, we manually curated more PPIs from the literature and developed a Naïve Bayesian based classifier to integrate and evaluate all the predicted PPIs, which made our database updated to AtPID 3.0 in 2008 as a rich source of information for system-level understanding of gene function and biological processes (2). In order to better serve the related research communities for the mechanism studies of various physiological activities, we annotated the Arabidopsis proteins in the AtPID 4.0 database with further information (e.g. functional annotation, subcellular localization, tissue-specific expression, phosphorylation information, SNP phenotype and mutant phenotype, etc.) and interaction qualifications (e.g. transcriptional regulation, complex assembly, functional collaboration, etc.) via further literature text mining and integration of other resources (3) (Table 1).

**Table 1.** A comprehensive comparison for the different versions of AtPID database

	Function annotation				Molecule interaction			
The version of AtPID	Protein functional description	Subcellular localization	Mutant	Phenotype annotation	Curated PPIs	Predicted PPIs	Curated transcriptional regulations	Predicted transcriptional regulations
AtPID 3 (2)	32 000	–	--	–	4666	23 396	–	–
AtPID 4 (3)	40 000	10 429	5121 mutants, 3431 genes	–	5565	98 174	8070	–
AtPID 5 (Current Version)	40 000	11 052	5609 mutants, 3916 genes	8202 mutant-PO associations	45 382	118 556	9435	31 991

Comparing with other organisms, plants have unique advantages on the mutagenesis and tissue culture, a large number of characterized stable *Arabidopsis* mutants have been reported in research literature, and large-scale seeds/mutant resources for plant functional studies were built for genome annotation and functional studies, e.g. uNASC Database (The European Arabidopsis Stock Centre), RAPID (RIKEN Arabidopsis Phenome Information Database), CSHL Database (the Arabidopsis Genetrap Website at Cold Spring Harbor Lab), Chloroplast Function Database, SeedGenes Database, AGRICOLA Database (Systematic RNAi knockouts in Arabidopsis), Araport (the Arabidopsis Information Portal) and TAIR (4–10). Mutant phenotypes are especially critical for functional studies of plants. Although great efforts have been made on collecting related data in plants, the mutant phenotypes are still largely under-annotated. AtPID has been committed to collect more mutants with significantly morphological alterations and tried to annotate all the mutants’ phenotypes in a systematical way. The Plant Ontology is a controlled vocabulary (ontology) that describes plant anatomy and morphology and stages of development for all plants (11). In order to index and annotate all the mutants in AtPID into a standard semantic framework, we cooperated with Shanghai Society for Plant Biology and annotated all the mutants to more specific downstream PO categories.

In this update, the AtPID 5.0 database greatly expands the information on PPIs, mutant phenotypes obtained from published literature (12–14), public databases and computational approaches. For mutant related information, the data of mutant phenotypes were carefully curated by biologists. In addition, novel associations between genes and phenotypes were predicted through Naïve Bayes method. Furthermore, we developed a more comprehensive visualization toolkit to view all the interactions at PPI, transcriptional regulation and genotype–phenotype levels under the same framework, which could easily show/map all other annotation information in our database for selected genes. All of the improvements and updates will accelerate researchers in exploiting information in our database in a more effective and comprehensive way.

## RESULTS

### Summary of new data in the updated AtPID 5.0

Comparing with the other well-used PPI resources (Table 2), the updated database indexed 45 382 curated PPIs and 118 556 predicted PPIs from literature mining, public databases or computational approaches. These numbers are significantly increased due to the ravenous growth and maturing biomedical national processing language and the large-scale experiments for functional studies (15–17). We also generated a comprehensive chloroplast proteomics dataset in Arabidopsis by large-scale proteomics experiments and indexed all 3134 credible chloroplast proteins into our annotation system. Furthermore, we systematically annotated 31 991 TFBS associations to 6891 genes based on the integration of expression profiling and cis-regulatory element information. This update largely enriches protein annotations in our database by tracking the recent research progresses of related areas and will greatly assist functional experiments and systematic studies.

**Table 2.** Numbers of interactions in AtPID 5.0 compared with the other well-used data resources

PPI-related database	Description for the PPI database	Curated PPIs	Predicted PPIs
AtPID 5.0	An integrated database resource for protein interaction network and functional annotation proteome.(http://www.megabionet.org/atpid)	45 382	118 556
PAIR	The predicted *Arabidopsis* interactome resource(http://www.cls.zju.edu.cn/pair/) (24)	5990	137 986
TAIR	A database of genetic and molecular biology data for the model higher plant *Arabidopsis thaliana*.(http://www.arabidopsis.org) (25)	6503	
BioGRID	An interaction repository with data compiled through comprehensive curation efforts.(http://thebiogrid.org/) (26)	42 216	
STRING	A database of predicted functional associations between proteins.(http://string-db.org/) (27)		>1 000 000

### Comprehensive annotation of genotype–phenotype associations

**Figure 1.**
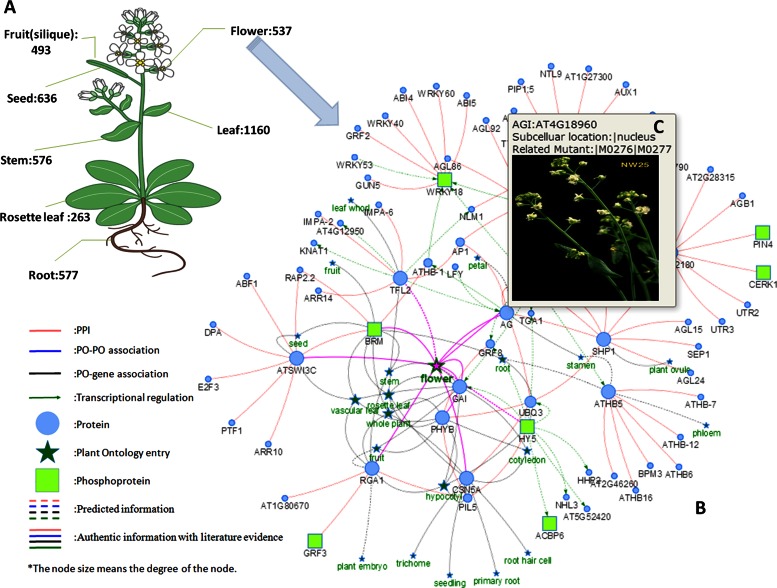
The overview and network display of the curated genotype–phenotype associations in AtPID 5.0. Top left corner (**A**) exhibits the top level Plant Ontology (PO) entries in *Arabidopsis* and the annotated gene numbers related to this PO. Bottom right corner (**B**) shows the flower-associated network. (**C**) Node with mouse hovering annotation.

Using text mining and database integration, the previous version (AtPID 4.1) collected 5121 mutants with significantly observable phenotypes related to 3431 genes. In the past few years, through in-depth cooperation with Shanghai Society for Plant Biology, we collected 488 new mutants and systematically annotated all the existed and new curated mutants’ phenotypes to 167 standardized plant ontology categories (Figure 1A). Comprehensive collection on phenotype data can help phenotype mechanism studies as what have been done in systematical exploration of disease associations (18,19). Strategies or algorithms have been developed to predict gene related functions by integrating multiple level data (18–20). We integrated three different information, PPIs, co-expression from expression profiling and GO annotation with Naïve Bayes method. PPIs were quantified by the extended Czekanowski–Dice distance (21) and missing values were complemented by orthologs in other 14 species’ experimental PPIs from STRING database (22). Shared Smallest Biological Processes (SSBPs) was applied to describe the possibility of gene interactions on GO annotation (23). Co-expression of gene pairs were computed over the microarrays mentioned above to predict regulatory interactions. The correlation coefficient values of the three information were low (PPIs-GO: 0.05; PPIs-co-expression: 0.08; co-expression-GO: −0.03), suggesting that features were independent from each other and satisfied with the assumption of Naïve Bayes method. Naïve Bayes was undertaken by e1071 package in R. The model showed high predictability, with average AUC 0.72. Finally, the prediction contains 4457 novel gene-PO pairs with 1369 genes, which could be a supplement to the known mutant information.

### User friendly visualization toolkit for comprehensive genotype–phenotype network

For the phenotype annotation information, we re-developed the network visualization application (Figure 1B and C) with JavaScript, which inherited all the functions of the old java applet, and added phenotype as a new node type. The new visualization application has better compatibility and performance due to the optimization of the database structure and the network generation methods. Meanwhile, it presents the network in a more interactive and comprehensive way. All the protein annotation information and protein relations in AtPID 5.0 can be presented simultaneously on the same view, and users can easily extend the network by double clicking any node on the border of current network. The combination of genotype–phenotype associations and the protein interaction information can provide existing knowledge of selected proteins to biologists in a very intuitive way and help them easily understand the functional relations to confirm their hypotheses or inspire them on new study designs.

## CONCLUSIONS

Here, we have made great efforts to provide a significantly improved resource for genotype–phenotype associations, which could serve as a resource for experimental design and facilitate genome-wide systematical studies in Arabidopsis. The AtPID 5.0 also provides illustrations of the functional annotation and protein network with a friendly web-based interface. We have largely extended the current annotation information by literature curation, bioinformatics predictions and also the high-throughput experimental data in the AtPID 5.0, e.g. we generated a comprehensive chloroplast proteomics dataset in Arabidopsis by large-scale proteomics experiments and indexed all the data as the evidence for subcellular localization in current AtPID. We will continue to accumulate more genome-wide data to better serve the research community.
